# How well do clinical prediction rules perform in identifying serious infections in acutely ill children across an international network of ambulatory care datasets?

**DOI:** 10.1186/1741-7015-11-10

**Published:** 2013-01-15

**Authors:** Jan Y Verbakel, Ann Van den Bruel, Matthew Thompson, Richard Stevens, Bert Aertgeerts, Rianne Oostenbrink, Henriette A Moll, Marjolein Y Berger, Monica Lakhanpaul, David Mant, Frank Buntinx

**Affiliations:** 1 Department of General Practice, KU Leuven, Kapucijnenvoer 33 blok J, 3000 Leuven, Belgium; 2 Department of Primary Care Health Sciences, University of Oxford, New Radcliffe House, Woodstock Road, Oxford, OX2 6GG, UK; 3 Erasmus MC - Sophia's Children Hospital, Dr Molewaterplein 60, 3015 GJ Rotterdam, The Netherlands; 4 Department of General Practice, University Medical Center Groningen, Hanze plein 1 Box 30001 9700RB Groningen, The Netherlands; 5 Department of General and Adolescent Paediatrics, University College London, Institute of Child Health, London, UK; 6 Research Institute Caphri, Maastricht University, PB 313, Nl 6200 MD, Maastricht, The Netherlands

**Keywords:** clinical prediction rules, serious infection in children, external validation, NICE guidelines feverish illness, Yale Observation Scale, diagnostic accuracy

## Abstract

**Background:**

Diagnosing serious infections in children is challenging, because of the low incidence of such infections and their non-specific presentation early in the course of illness. Prediction rules are promoted as a means to improve recognition of serious infections. A recent systematic review identified seven clinical prediction rules, of which only one had been prospectively validated, calling into question their appropriateness for clinical practice. We aimed to examine the diagnostic accuracy of these rules in multiple ambulatory care populations in Europe.

**Methods:**

Four clinical prediction rules and two national guidelines, based on signs and symptoms, were validated retrospectively in seven individual patient datasets from primary care and emergency departments, comprising 11,023 children from the UK, the Netherlands, and Belgium. The accuracy of each rule was tested, with pre-test and post-test probabilities displayed using dumbbell plots, with serious infection settings stratified as low prevalence (LP; <5%), intermediate prevalence (IP; 5 to 20%), and high prevalence (HP; >20%) . In LP and IP settings, sensitivity should be >90% for effective ruling out infection.

**Results:**

In LP settings, a five-stage decision tree and a pneumonia rule had sensitivities of >90% (at a negative likelihood ratio (NLR) of < 0.2) for ruling out serious infections, whereas the sensitivities of a meningitis rule and the Yale Observation Scale (YOS) varied widely, between 33 and 100%. In IP settings, the five-stage decision tree, the pneumonia rule, and YOS had sensitivities between 22 and 88%, with NLR ranging from 0.3 to 0.8. In an HP setting, the five-stage decision tree provided a sensitivity of 23%. In LP or IP settings, the sensitivities of the National Institute for Clinical Excellence guideline for feverish illness and the Dutch College of General Practitioners alarm symptoms ranged from 81 to 100%.

**Conclusions:**

None of the clinical prediction rules examined in this study provided perfect diagnostic accuracy. In LP or IP settings, prediction rules and evidence-based guidelines had high sensitivity, providing promising rule-out value for serious infections in these datasets, although all had a percentage of residual uncertainty. Additional clinical assessment or testing such as point-of-care laboratory tests may be needed to increase clinical certainty. None of the prediction rules identified seemed to be valuable for HP settings such as emergency departments.

## Background

Acute infection is the most common presentation in children attending settings of ambulatory care (AC) [1,2]. Although most infections are self-limiting, they remain an important cause of morbidity and mortality in children in economically developed countries [3-5]. In the UK, infections account for 20% of childhood deaths, especially in children under 5 years of age [6]. Serious infections in children are usually defined as sepsis (including bacteremia), meningitis, pneumonia, osteomyelitis, cellulitis, and complicated urinary-tract infection (UTI; positive urine culture combined with systemic features such as fever) [3]. As a result of immunization against *Haemophilus influenzae *and *Streptococcus pneumoniae*, the incidence of these diseases has decreased steadily over recent decades, and they are now estimated to account for less than 1% of all acute childhood infections in primary care (PC) [2,7].

The combination of low incidence, non-specific initial clinical presentation, and potential for rapid deterioration makes the assessment of acutely ill children difficult [8,9]. Clinical prediction rules (CPRs) and guidelines may assist in the early recognition of serious infections [3]. In a previous systematic review, we identified all available CPRs (seven in total), based on signs and symptoms, for identifying any serious infection (two rules), pneumonia (two), meningitis (two), and dehydration from gastroenteritis (one rule) in AC settings [3]. Four of these seven CPRs were derived for use in emergency-care settings and their applicability in PC and AC settings has not been confirmed.

Only one rule, the Yale Observation Scale (YOS) [10] has been prospectively assessed in four studies [11-14], of which only two assessed the YOS in the intended age group of 3 to 36 months [12,14]. We also identified two national guidelines for the assessment of feverish children (Guideline on Feverish Illness in Children by the National Institute for Health and Clinical Excellence (NICE) [15] and the guidelines from the Dutch College of General Practitioners (NHG) [16]). A focused literature search identified an additional CPR published after this review: an emergency-department (ED) rule [17] to diagnose pneumonia, UTI, or bacteremia (see Additional file 1).

Although some of these guidelines (NICE guidelines, NHG alarm symptoms) are often used in clinical practice, very little external validation to support their use in practice has been performed in new and independent populations [18]. This raises questions about the robustness of the rules and their generalizability.

The aim of this study was to examine the diagnostic accuracy both of the CPRs identified by the systematic review and of the evidence-based guidelines, using retrospective external validations on individual patient datasets from ambulatory pediatric settings including PC and ED settings from three European countries.

## Methods

### Identification of datasets

**Figure 1:**
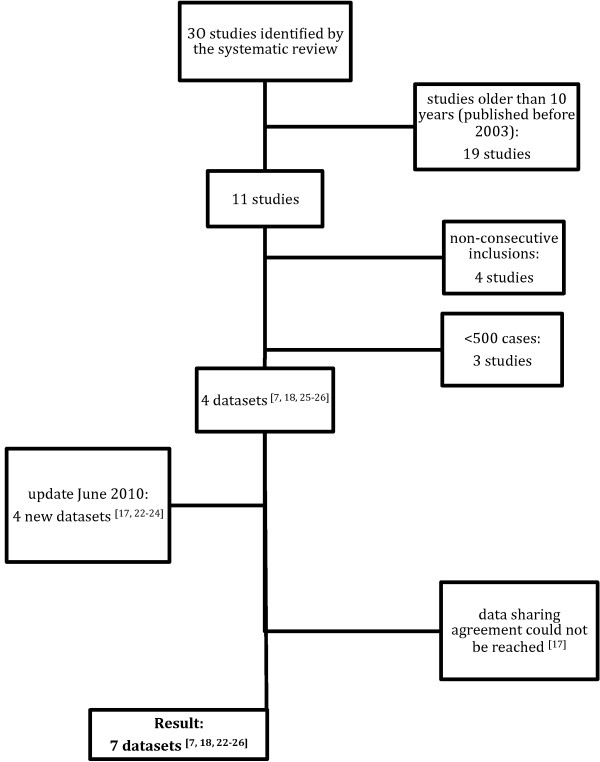
**Flowchart of dataset inclusion**.

We included datasets from studies identified in the systematic review [3], which had been published within the past 10 years, and from expert contacts. The criteria used to select datasets (Table 1), were design (cohort studies that enrolled children consecutively), sample size (> 500 children), participants (children aged 0 to 18 years or subgroups of these), setting (AC defined as general or family practice, pediatric outpatient clinics, pediatric assessment units, or EDs in developed countries), outcome (serious infection), and data availability (agreement to share data) (Figure 1).

**Table 1:** Criteria for inclusion and exclusion of datasets in validation analysis.

Characteristic	Inclusion	Exclusion
Publication date	Studies published in the past 10 years	Studies published before 2003

Design	Studies that had recorded clinical features; prospective or retrospective cohort study design	Unclear methods

Sample Size	> 500 children	< 500 children

Participants	Age between 1 month and 18 years of age; studies including children spanning this age range were included if they reported age (or age could be calculated)	Children with congenital or obtained immunodeficiency. Age outside the required range

Setting	Ambulatory care (defined as general or family practice, pediatric outpatient clinics, pediatric assessment units, or emergency departments). Developed countries, defined using the United Nations list, which included Europe, Canada, USA, Australia, New Zealand and Japan	Studies conducted in developing countries

Outcome	Serious infection, defined as sepsis (including bacteremia), meningitis, pneumonia, osteomyelitis, cellulitis, and complicated urinary-tract infection (positive urine culture and systemic effects such as fever)	Diagnosis other than serious infection

Data availability	Agreement to share data	

### Ethics approval

This research conformed to the Helsinki Declaration and to local legislation. The study authors, agreeing to share data, obtained ethics approval from their regional research ethics committees before the study for the initial data collection of the included datasets.

### Processing of included datasets

Direct access to the raw data of each dataset was granted and key characteristics of each of the datasets were extracted (Table 2). The variables used in each dataset were translated to English if necessary, and the translation, coding, and definition of variables were clarified with the authors of the relevant study.

**Table 2:** Characteristics of datasets used for external validation of prediction rules.

Dataset	Setting	Country	N	Age, years; mean (range)	Prevalence ofserious infection % (95% CI)	Inclusion criteria	Exclusion criteria
Van den Bruel *et al*. 2007 [7]	GP/AP/ED	BE	4102	5.0 (0.0 to 16.9)	0.8 (0.5 to 1.1)	Children ≤16 years with acute illness max 5 days	Traumatic or neurological illness, intoxication, psychiatric or behavioral problems without somatic cause or an exacerbation of a chronic condition. No repeated inclusion of same infant within 5 days. Exclusion of physicians if the assumption of consecutive inclusion was probably violated

Roukema *et al*. 2008 [24]	ED	NL	1750	2.9 (0.1 to 15.7)	12.3 (10.8 to 13.9)	All children with fever (>38°C) at ED, without meningeal irritation	Chronic disease, Immunodeficiency

Bleeker *et al*. 2007 [25]	ED	NL	595	0.9 (0.0 to 3.0)	23.0 (19.6 to 26.4)	Children with fever T>38°C at ED, no clear focus identified after evaluation GP of history by pediatrician	Chronic disease, Immunodeficiency

Monteny *et al*. 2008 [22]	GP	NL	506	2.2 (0.3 to 5.9)	4.0 (2.3 to 5.7)	Children aged 3 months to 6 years, contacting a GP cooperative after hours with fever as the presenting symptom	Language barriers, no repeated inclusion within the previous two weeks

Brent *et al*. 2011 [23]	ED	UK	2777	3.3 (0.0 to 18.4)	5.3 (4.5 to 6.1)	All children presenting with a medical problem to the pediatric emergency-care unit whatever their age	Children who required immediate resuscitation. Comorbidity and chronic illness

Thompson *et al*. 2009 [18]	PAU	UK	700	4.6 (0.0 to 16.0)	37.7 (34.1 to 41.3)	Children aged 3 months to 16 years with suspected acute infection	Children with diseases liable to cause repeated serious bacterial infection, and infections resulting from penetrating trauma

Oostenbrink *et al*. 2004 [26]	ED	NL	593	3.7 (0.1 to 16.1)	43.8 (39.8 to 47.9)	Children aged 1 month to 16 years, meningeal signs at GP, pediatrician-referred or self-referred with neck pain	Comorbidity, ventriculoperitoneal drain

AP, ambulatory pediatric care; BE, Belgium; CI, confidence interval; ED, emergency department; GP, general practice; NL, the Netherlands; PAU, pediatric assessment unit; UK, United Kingdom.

We used the following criteria to determine which dataset could be used to validate each CPR and guideline, and which diagnoses should be included in the composite outcome of serious infection.

• Datasets used to derive a CPR were not used to validate the same rule.

• When variables were not entirely identical with the variables of the original CPR, we identified proxies where possible. For example, the variable 'dyspnea' of the five-stage decision tree (FSDT) and the pneumonia rule was not recorded in three datasets; we therefore used either 'respiratory distress' or 'chest flaring' as a proxy (for a full list of all approximations, see Additional file 2 and Additional file 3).

• Based on the number of required variables, whenever one-third or more (fever guidelines), one or more (pneumonia rule, meningitis rule) or two or more (YOS, FSDT) of the required variables were not recorded, that dataset was not used for validation of that specific rule. We performed sensitivity analyses as described below.

• Missing data on variables used in the validation were not imputed because the necessary missing-at-random assumption was likely to be incorrect because some of the datasets consisted of routinely collected data from medical records.

• Apart from the approximations used (see Additional files 2, Additional file 3), no alterations of the original data were performed. We report the number of observations available for analysis of each prediction rule after applying these assumptions.

• In contrast to the other dichotomous rules, the YOS generates a sum score. We defined an abnormal result using two pre-selected cut-offs (of 8 or 10).

• Serious infection was defined as sepsis (including bacteremia), meningitis, pneumonia, osteomyelitis, cellulitis, or complicated UTI [3]. These diagnoses were available for all datasets, and assessment of the diagnoses to ensure comparability of outcomes was discussed with the authors of each study.

The settings in the included datasets were stratified as having low prevalence (LP; 0 to 5%), intermediate prevalence (IP; 5 to 20%) or high prevalence (HP; >20%) of the serious infection(s) of interest (including all serious infections, pneumonia, meningitis) with the clinical assumption that diagnostic goals are different in each setting. In LP settings, CPRs should have high sensitivity in order to correctly rule out (at a negative likelihood ratio (NLR) of up to 0.2) the target disorder(s) at a reasonable cost in terms of referral or admission rates [19,20].

The accuracy of the CPRs was assessed retrospectively in each of the available prospectively collected datasets by calculating sensitivity, specificity, predictive value, and likelihood ratio (LR). We used dumbbell plots to display the change from pre-test to post-test probabilities [3].

To avoid the risk of influencing diagnostic accuracy by either an arbitrarily chosen number of required variables, or the age range available in each dataset compared with the intended age range of the rule, we performed the following sensitivity analyses after obtaining initial results with the different CPRs. Firstly, when a CPR was specifically designed for a certain age group (, for example, the YOS for children aged 3 to 36 months and the NICE guidelines for children up to 5 years of age), we compared the 95% confidence intervals (CIs) of the diagnostic characteristics (sensitivity, specificity, LRs and area under the curve (AUC)) [21] in the target age group with the entire age range of the dataset at hand. Second, when one or more variables of the original prediction rule were missing, we examined those same diagnostic characteristics in the datasets with no missing variables, to avoid biasing results on the number of missing variables. Whenever more than one (for the CPRs) or more than two (for the fever guidelines) original variables were missing, we did not perform sensitivity analysis, based on the rationale that missing two (or more) of a maximum of six variables (for the CPRs) or three (or more) of a maximum of eight original variables (for the fever guidelines) did not seem clinically sensible. This was discussed and confirmed by all study authors, contributing data to the current study.

Meta-analysis of the pooled results of the multiple external validations was not possible because substantial clinical heterogeneity was found in these datasets, including differences in setting, inclusion criteria, immunization schedules, and definition of serious infection. Additionally, the small number of included studies would have led to a high level of uncertainty in the estimates of the variances of the random effects for both the bivariate and hierarchical summary receiver operating characteristic models, if heterogeneity were to be explored statistically. Inclusion or exclusion of a single study would affect the convergence of the model greatly [21]. The individual patient data were analyzed in every dataset separately. The translation, re-coding, and data checking were performed by one author (JV), and the results of each step were discussed with all of the other authors. All analyses were performed with Stata software (version 11.2; Stata Corp., College Station, TX, USA).

## Results

### Included datasets

We obtained seven datasets providing data on 11,023 children: two LP datasets from general practice [7,22], two IP datasets from EDs [23,24] and three HP datasets from EDs [25,26] or pediatric assessment unit s[18] in the UK (n = 2), the Netherlands (n = 4) and Belgium (n = 1) (Figure 1, Table 2). Children were included based on presence of fever [22,24,25], acute illness [7,23], or acute infection [18], or on referral for meningeal signs [26]. Children with various co-morbidities were excluded in six studies, and one study excluded children who required immediate resuscitation. The outcome in all studies included sepsis, meningitis, pneumonia, and complicated UTI as part of the outcome variables. Osteomyelitis and cellulitis were explicitly mentioned in five and three datasets, respectively. The mean age ranged from 0.94 to 5.0 years, and prevalence of serious infection ranged from 0.8 to 43.8%.

### Clinical predictors included in the datasets

Most datasets included basic demographic characteristics such as age, duration, and severity of illness, as well as referral status. Temperature was recorded in all datasets (with missing data rates ranging from 0 to 18%), heart rate in five datasets (missing in 2 to 48%), capillary refill time in five (missing in 2 to 48%), respiratory rate in four (missing in 15 to 53%), and oxygen saturation in four (missing in 4 to 74%).

Validation of the FSDT [7] was possible in five datasets [18,22-25], of which four had all variables present using 'clinical sick impression' as a proxy for 'physician's gut feeling that something is wrong', and 'respiratory distress' or 'chest flaring' as a proxy for 'dyspnea' (See Additional file 2). Because the variable 'diarrhea' was missing in one dataset [25], we performed a sensitivity analysis comparing the results of the four remaining variables, as noted below.

Five datasets [18,22-25] were available for one pneumonia rule [7], developed in PC settings, with 'sick impression to clinician' as a proxy for the 'physician's gut feeling that something is wrong' and 'nasal flaring' for 'dyspnea'. A second pneumonia rule, derived in the same dataset [7], which included 'respiratory distress' and 'parental concern the illness is different' could not be validated, as the latter variable was not recorded in any of the validation datasets.

A meningitis rule, derived by Offringa *et al*. [27] for children in the ED, was validated in three datasets [7,18,26]. Because all items except 'nuchal rigidity' were present in one additional dataset [23], we performed a sensitivity analysis comparing the results of the two remaining variables, eventually excluding this dataset from the analysis, as noted below. A second meningitis rule could not be validated because the absence of its key variables in these datasets [28].

For the YOS [10], developed in secondary care, three datasets had recorded variables used in the original Yale scoring [18,22,23] (see Additional file 2). Because the YOS item 'reaction to parent stimulation' was missing in one dataset [29], we performed a sensitivity analysis comparing the results of the five remaining YOS items, as noted below. None of the datasets included sufficient variables to validate the prediction rule to identify gastroenteritis with dehydration developed by Gorelick *et al*. [30], or the prediction rule developed by Craig *et al*. [17].

The NICE guideline for feverish illness in children and the NHG alarm symptoms [15,16] were validated in four [18,22,23,26] and five [7,18,22,23,26] datasets, respectively.

### Validation results

**Figure 2:**
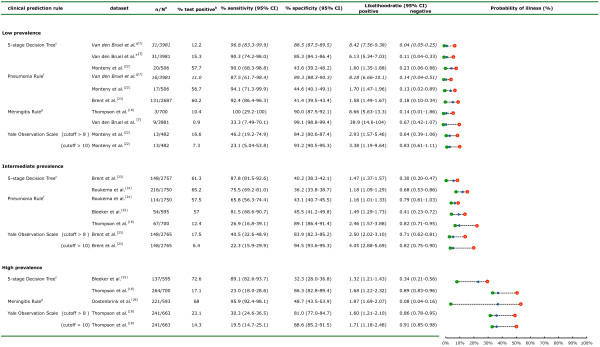
**Results of external validation of clinical prediction rules (CPRs) to rule in or rule out serious infection**. ^a^Number of cases (n) out of the total population of all children (N). ^b^Percentage testing positive in all included children. ^c^If yes to any of five sequential questions: 1) clinical instinct that something is wrong, 2) dyspnea, 3) temperature greater than 39.5°C, 4) diarrhea, 5) age 15 to 29 months; ^d^Derivation study (italic). ^e^'clinical instinct that something is wrong' replaced by 'clinical impression'. ^f^If yes to any of the following: 1) shortness of breath, 2) clinicians concern. ^g^If yes to any of the following: 1) petechiae, 2) nuchal rigidity, 3) coma; probability of illness (in percentage) before testing (blue dot), after a positive test result (red dot with plus to sign) and after a negative test result (green dot with minus to sign).

**Figure 3:**
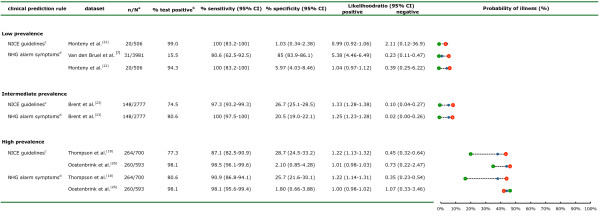
**Results of external validation of the evidence-based clinical guidelines for management of fever**. ^a^Number of cases (n) out of the total population of all children (N). ^b^Percentage testing positive in all included children. ^c^'Traffic light' system of clinical features that are designed to be used to assess the risk of serious infection, and to provide clinical guidance for actions needed according to these categories. ^d^Alarm symptoms at clinical examination: seriously ill impression, reduced consciousness, persistent vomiting, petechiae, tachypnea and/or dyspnea, reduced peripheral circulation, pallor, and signs of meningeal irritation; probability of illness (in percentage) before testing (blue dot), after a positive test result (red dot with + sign) and after a negative test result (green dot with - sign).

The characteristics of diagnostic accuracy, according to prevalence, are shown for all CPRs (Figure 2, Figure 3).

### Low-prevalence settings

The FSDT had a sensitivity of 90% (95% CI 68 to 99%) and an NLR of 0.2 (95% CI 0.1 to 0.9) in the single LP dataset available for validation, with false-positive test results (for example, no serious infection present) in 54% of all children examined [22]. The sensitivities of the pneumonia rule were 94% (95% CI 71 to 100%) and 92% (95% CI 86 to 96%) in two datasets, with NLRs of less than 0.2, resulting in 54% and 56% false-positive test results [22,23]. Validation of the meningitis rule in two LP datasets [7,18] resulted in sensitivities ranging from 33% (PC dataset) to 100% (secondary care dataset with a LP for meningitis) with NLRs ranging from 0.1 to 0.7. The YOS, with cut-offs of 8 and 10, provided sensitivities below 46% in one LP dataset [22], but had an NLR of greater than 0.6. The NICE 'traffic light' system with any amber or red sign present, and the NHG alarm symptoms were extremely sensitive (100%) with NLR of greater than 0.4, testing as false positive in 90 to 95% of all children in one LP dataset [22].

### Intermediate-prevalence settings

The FSDT provided moderate sensitivities of 76% (95% CI 69 to 81%) and 88% (95% CI 82 to 93%), in two IP settings [23,24] (with NLR ranging from 0.3 to 0.7). The pneumonia rule had sensitivities ranging from 66 to 82% in two datasets [24,25] but in a third dataset [18] with the highest prevalence (11%) of pneumonia, the sensitivity was only 27% (95% CI 17 to 39%) and the NLR was 0.8 (95% CI 0.7 to 1.0). The YOS, with cut-off values of 8 and 10, provided sensitivities of less than 41% in one IP dataset [23], and had an NLR greater than 0.7. Finally, the NICE guideline and NHG alarm symptoms had high sensitivity (97 to 100%) in one IP setting [23] with NLR of less than 0.1.

### High-prevalence settings

In one HP setting [25], the FSDT had a sensitivity of 89% (95% CI 83 to 94%) with NLR of 0.4 (95% CI 0.2 to 0.6). However, sensitivity was only 23% (95% CI 18 to 29%) with NLR of 0.8 (95% CI 0.7 to 0.9) in a pediatric assessment unit [18]. In one study [26] that included children with meningeal signs identified by the referring physician, the meningitis rule showed high sensitivity, at 96% (95% CI 92 to 98%) and NLR of 0.1 (95% CI 0.04 to 0.2). The Yale score, with cut-offs of 8 and 10, provided sensitivities of less than 30% in one HP dataset [18], and NLR of 0.9. Finally, both NICE guideline and NHG alarm symptoms had sensitivities ranging from 87 to 99% in two HP datasets [18,26] with NLR greater than 0.4.

### Sensitivity analyses

Comparing the 95% CIs, we found similar results for the diagnostic characteristics of the YOS and the NICE guidelines in children of all ages as well as in children for whom the rules were originally designed (3 to 35 months and up to 5 years, respectively) (see Additional file 4).

Comparing the results of the datasets in which the complete prediction rule could be validated with those of the datasets with one or two missing variables (five items of the YOS, four items of the FSDT, and six items of the NHG alarm symptoms), all diagnostic characteristics were found to be similar through comparison of the 95% CIs (see Additional file 4).

By contrast, dropping 'nuchal rigidity' from the meningitis rule resulted in a lower sensitivity (67% (95% CI 9 to 99%) versus 100% (95% CI 29 to 100%) when all three variables were considered) in one dataset [18], eliminating one additional dataset, which had only two out of three original variables available, for further use in the validation [23].

## Discussion

### Main findings

None of the CPRs examined in this study provided perfect diagnostic accuracy. The best performing CPR for ruling out serious infection in an LP setting was the FSDT, which uses the physician's gut feeling, the patient's age and temperature, and presence of dyspnea and diarrhea [7]. Sensitivity was lower than that reported in the original study, possibly explained by our use of 'clinical impression' as a proxy for 'physician's gut feeling' which has been reported to be of lower diagnostic value [3].

Both the NICE guideline and the NHG alarm symptoms high sensitivity in both LP and IP settings, suggesting possible clinical value for ruling out serious infections in children presenting in these settings. However, large numbers of children were flagged as potentially having a serious infection. If the prediction rules were to be used in clinical practice, additional clinical assessment, additional testing, or review at a later stage would be necessary to avoid inappropriate referrals or hospital admissions.

For the well-known YOS, all sensitivities were low, which is similar to the results of a previously reported pooled sensitivity based on the meta-analysis of seven studies [3].

Other disease-specific rules (pneumonia and meningitis) had acceptable sensitivities only in the LP settings, indicating value as rule-out tests. However, the percentage of false positives was too high in all datasets, apart from one IP dataset, probably due to the higher prevalence of pneumonia in this dataset [18].

### Limitations

Despite the large number of datasets available, we were able to validate only four of the eight prediction rules plus both guidelines. The methodological challenges encountered in performing these retrospective validations in prospectively collected datasets limit the translation into clinical practice. Performance of prediction rules was generally lower than in their original derivation studies. One possible explanation for this is the approximations that we used for variables measured and recorded in different ways (and different languages).

To avoid potential bias from validating in datasets that were missing variables, a sensitivity analyses was performed and, if findings were robust throughout the different validation datasets, subsequent validation was deemed suitable. In addition to variation in recorded variables, multiple other sources of heterogeneity were found in the included databases, including differences in setting, inclusion criteria, immunization schedules, and definition of serious infection.

### Strengths

Although the limitations may be substantial, this is the first study to externally validate existing CPRs in different types of clinical settings. We used individual patient data from a total of seven existing datasets comprising 11,023 children presenting to PC or EDs in three European countries to retrospectively validate existing prediction rules and national evidence-based guidelines. Previously, only a single prediction rule had been prospectively validated in external datasets [11-14]. Our study therefore presents the first robust attempt to simultaneously validate multiple current prediction rules and evidence-based guidelines for management of one of the most common clinical conditions in AC settings. We anticipate that our findings will be applicable to guideline developers worldwide.

### Comparison with other studies

The YOS was initially developed to identify serious illness in febrile children aged 3 to 36 months, but was subsequently discarded based on three prospective validation studies (of which only one was carried out in the intended age group) [11,13,14]. The rule was also used to stratify patients in five studies evaluating inflammatory markers (such as procalcitonin and C-reactive protein), with discouraging results [31-35]. Bang *et al*. reported a slightly better performance of the YOS in predicting bacteremia in febrile children in an HP study (28%), which does not apply to most AC settings [12]. Although the YOS was not useful for ruling out a serious infection in our analysis, a score of greater than 10 (with a combination of the presence of abnormal color or hydration status, failure to respond to parents, different cry, and abnormal sleepiness) did slightly increase the likelihood of a serious infection in these datasets.

### Clinical implications

With decreasing incidence of serious infections, clinicians will increasingly rely on CPRs in practice, particularly in high-volume triage settings. In these settings, 'generic' rules, which apply to all serious infections, are more useful than disease-specific rules. Particularly in settings where diagnosis of serious illness in children is essential (for example, PC), the FSDT, the NICE guidelines, and the NHG alarm symptoms may be used to rule out serious infections in a large proportion of children. We suggest that the FSDT, mainly consisting of the child's breathing status and temperature and the clinician's gut feeling that something is wrong, should be used for assessment of every acutely ill child. The meningitis rule, with absence of nuchal rigidity, petechiae, and coma, indicate that meningitis is highly unlikely in LP settings.

Clinicians should be aware that none of the CPRs provide perfect discrimination, and it is perhaps unrealistic to expect such rules to provide this. Residual uncertainty may be further improved by conducting more detailed clinical assessments, repeating the assessment after some time, using additional testing (for example, urine or blood tests), and in most cases, providing an appropriate safety netting advice for children sent home detailing instructions on when to seek further care [36].

### Research implications

Most CPRs never undergo further validation or are implemented, perhaps inappropriately, with insufficient external validation [37,38]. Indeed very few CPRs for the identification of children with serious infection have undergone either extensive validation or formal impact analysis, limiting the ability to truly evaluate their performance and to balance benefits and harms [19,39]. In general, CPRs perform worse when validated in new populations [40].

Our study presents the first multiple external validation of CPRs in this common clinical area, and identifies which of them offer the best diagnostic accuracy in different types of clinical settings. This illustrates the clear need to perform extensive prospective validation and impact analysis of CPRs prior to clinical implementation [39,41]. The FSDT and the NICE guidelines for assessment of feverish children are potential candidates for future prospective validation studies examining their performance in new prospectively collected data on similar populations.

We recognize the previously identified major mismatch [3], between the clinical settings where the majority of children with acute infections seek help (that is, PC), and the number of studies performed in that setting (two studies) (Table 2). There is a pressing need for more studies conducted in PC or in LP ED settings to validate CPRs for serious infection, or the need for hospital referral/admission. Given the relative infrequency of serious infections, such studies need to include large cohorts of children [7,8]. CPRs are mostly designed to rule out serious infections, often at the expense of moderate to low ability for inclusion. As no rule is perfect at ruling out infection, research on the most effective content and methods of delivery with appropriate safety netting advice in PC and EDs is essential [8,36,42]. Adding newer tests such as point-of-care inflammatory markers may improve the diagnostic value of these rules, but the performance of these markers in non-referred populations has to be tested [43].

## Conclusions

None of the CPRs examined in this study provided perfect diagnostic accuracy. In LP settings (for example, PC) or IP settings, prediction rules, such as the FSDT and evidence-based guidelines (NICE guideline and the NHG alarm symptoms) had high sensitivity, providing promising rule-out value for serious infections in these datasets, although all seemed to leave residual uncertainty. Additional clinical assessment or testing such as point-of-care inflammatory markers may be needed to increase clinical certainty. None of the prediction rules identified seemed to be valuable for HP settings (for example, EDs).

## List of Abbreviations

CPR: clinical prediction rule; ED: emergency department; HP: high prevalence; IP: intermediate prevalence; LP: low prevalence; NLR: negative likelihood ratio; AC: ambulatory care; UTI: urinary tract infection; FSDT: five-stage decision tree; NHG: 'Nederlands Huisartsen Genootschap'; NICE: National Institute for Health and Clinical Excellence; PC: primary care; YOS: Yale Observation Scale.

## Competing interests

All authors declare they have no competing interests. The study sponsor had no role in study design, in the collection, analysis, or interpretation of data, in the writing of the report, or in the decision to submit the paper for publication.

## Authors' contributions

JV undertook the translation, the synopsis, the re-coding and the data checking and the results of each step were discussed with all study authors (AVdB, MB, RO, HM, MT, ML), contributing data to the current study. JV undertook the external validation analysis and drafted the report. AVdB, MT, RS, BA, RO, HM, MB, ML, DM, and FB conceived the analyses, co-drafted the report, and commented on it. All authors have read and approved the final manuscript.

## Pre-publication history

The pre-publication history for this paper can be accessed here:

http://www.biomedcentral.com/1741-7015/11/10/prepub

## Supplementary Material

Additional file 1 **Details of the clinical prediction rules identified in the systematic review**. CRT, capillary refill time; RR, respiratory rate; Temp, temperature. Click here for file

Additional file 2 **variables and proxies used for validation of clinical prediction rules**. N = Number of children in dataset; % n/N = Percentage of cases (n) out of all children (N) used for the external validation analysis; green font indicate original variable, red font,variable not recorded, blue font, proxy variable. ^a^Derivation study (italic). ^b^'Clinical sick impression' used as proxy for 'physician's gut feeling that something is wrong', ^c^'Respiratory distress' used as proxy for 'dyspnoea'. ^d^'Chest flaring' used as proxy for 'dyspnoea'. ^e^'Meningeal irritation' used as proxy for 'nuchal rigidity'. ^f^'Unconsciousness' used as proxy for 'coma'. Click here for file

Additional file 3 **Variables and proxies used for fever guidelines validation**. N. number of children in dataset; % n/N. percentage of cases (n) out of all children (N) used for the external validation analysis; green font. original variable Click here for file

Additional file 4 **Sensitivity analyses**. CI, confidence interval; underlined, 95% CIs not comparable. Click here for file
