# Supplementary material for: How well do clinical prediction rules perform in identifying serious infections in acutely ill children across an international network of ambulatory care datasets?
Source: BMC Med. 2013 Jan 15;11:10. doi: 10.1186/1741-7015-11-10 (PMC3566974; doi:10.1186/1741-7015-11-10)
Supplement: Additional file 1 — Details of the clinical prediction rules identified in the systematic review. CRT, capillary refill time; RR, respiratory rate; Temp, temperature. [file 1741-7015-11-10-S1.DOC]

# Additional file 1: Details of the clinical prediction rules and guidelines identified in the systematic review

| Name of Clinical Prediction Rule | Clinical features | | | | | | | | | | | | | Derivation study |
| --- | --- | --- | --- | --- | --- | --- | --- | --- | --- | --- | --- | --- | --- | --- |
| All serious infections |  | | | | | | | | | | | | |  |
| Yale Observation Scale | Quality of Cry | Reaction to parents’ stimulation | | | State Variation | | | | Colour | | Hydration | Response to social overtures | | McCarthy et al. |
| Values | Strong OR not crying 1  Whimpering 3  Weak 5 | Cries briefly 1  Cries off and on 3  Continual cry 5 | | | Stays awake 1  Awakes with stimulation 3  Falls to sleep 5 | | | | Pink 1  Pale extremities 3  Pale OR cyanotic 5 | | Skin normal 1  Dry mouth 3  Skin doughy 5 | Smiles OR Alerts 1  Brief smile OR alerts briefly 3  No smile OR face anxious 5 | |  |
|  | Calculate the sum of all six feature values (cut-offs used in literature: 8, 9 or 10) | | | | | | | | | | | | |  |
| Five Stage Decision Tree | Clinician instinct that something is wrong | | | Dyspnoea | | | Temperature > 39.95°C | | | Diarrhoea | | | Age 15-25 months | Van den Bruel et al. |
| Values | No 0  Yes or unknown 1 | | | No or unknown 0  Yes 1 | | | <39.95°C 0  >39.95° C 1 | | | No or unknown 0  Yes 1 | | | No or unknown 0  Yes 1 |  |
|  | If yes to any of these five features | | | | | | | | | | | | |  |
| Pneumonia |  | | | | | | | | | | | | |  |
| Pneumonia Rule n°1 | Parental concern illness is different | | | | | | | Shortness of breath | | | | | | Van den Bruel et al. |
| Values | If yes to any of these two features | | | | | | | | | | | | |  |
| Pneumonia Rule n°2 | Clinician concern illness is different | | | | | | | Shortness of breath | | | | | | Van den Bruel et al. |
| Values | If yes to any of these two features | | | | | | | | | | | | |  |
| Meningitis |  | | | | | | | | | | | | |  |
| Meningitis Rule n°1 | Any abnormal neurological finding | | | | | | | Sought care < 48hrs | | | | | | Offringa et al.[27] |
| Values | If yes to any of these two features | | | | | | | | | | | | |  |
| Meningitis Rule n°2 | Petechiae | | | Nuchal rigidity | | | | | | Coma | | | | Joffe et al.[28] |
| Values | If yes to any of these three features | | | | | | | | | | | | |  |
| Gastroenteritis with dehydration |  | | | | | | | | | | | | |  |
| Gastroenteritis Rule n°1 | Absent tears | | Dry mucous membranes | | | Ill appearance | | | | | Poor peripheral circulation | | | Gorelick et al.[30] |
| Values | If yes to any two of these four features | | | | | | | | | | | | |  |

| Name of guideline | Clinical features | | | | | | | | | | Derivation study |
| --- | --- | --- | --- | --- | --- | --- | --- | --- | --- | --- | --- |
| Fever guidelines | | | | | | | | | | |  |
| NICE traffic light system | Colour | | Activity | | Respiratory | | Hydration | | Other | | NICE: Feverish Illness in Children[15] |
| Amber traffic lights | - pallor | | - not responding to social cues  - wakes only with prolonged stimulation  - decreased activity  - no smile | | - nasal flaring  - tachypnoea  (age 6-12 months: RR  >50/min; age >12  months: > 40/min)  - O_2_ saturation ≤ 95%  - crackles | | - dry mucous membranes  - poor feeding in infants  - CRT ≥ 3 seconds  - reduced urine output | | - fever for temp ≥5 days  - swelling of a limb or joint  - non-weightbearing limb/not using  extremity  - a new lump >2 cm | |  |
| Red traffic lights | - pale/mottled/  ashen/blue | | - no response to social cues  - appears ill to doctor  - does not wake or if roused does not stay  awake  - weak high-pitched or continuous cry | | - grunting  - tachypnoea (>60/min)  - moderate/severe chest indrawing | | - reduced skin turgor | | - age 0-3 months, temp ≥38°C  - age 3-6 months, temp ≥39°C  - non-blanching rash  - bulging fontanelle  - neck stiffness  - status epilepticus  - focal neurological signs  - focal seizures  - bile-stained vomiting | |
|  | |
| Values | If yes to any of these 5 categories, each scoring 2 to 13 features | | | | | | | | | |  |
| NHG alarm symptoms | Seriously ill | Disturbed consciousness | | Persistent vomiting | Petechiae | Tachypnoea or dyspnoea | | Reduced peripheral circulation | Pallor or ashen or blue | Meningeal irritation | Dutch College of General Practitioners (NHG): Feverish Illness in Children [16] |
| Values | If yes to any of these eight features | | | | | | | | | |  |
